# BAFF Controls Neural Cell Survival through BAFF Receptor

**DOI:** 10.1371/journal.pone.0070924

**Published:** 2013-07-29

**Authors:** Satoru Tada, Teruhito Yasui, Yuji Nakatsuji, Tatsusada Okuno, Toru Koda, Hideki Mochizuki, Saburo Sakoda, Hitoshi Kikutani

**Affiliations:** 1 Department of Neurology, Graduate School of Medicine, Osaka University, Suita, Osaka, Japan; 2 Department of Molecular Immunology, Research Institute for Microbial Disease, WPI Immunology Frontier Research Center (IFReC), Osaka University, Suita, Osaka, Japan; 3 Department of Neurology, National Toneyama Hospital, Toyonaka, Osaka, Japan; Kyushu University, Japan

## Abstract

Various neuroprotective factors have been shown to help prevention of neuronal cell death, which is responsible for the progression of neurodegenerative diseases such as amyotrophic lateral sclerosis (ALS). However, most of these therapeutic potentials have been tested by administration of recombinant proteins, transgenic expression or virus vector-mediated gene transfer. Therefore, it remains to be clarified whether any endogenous factors has advantage for neuroprotection in a pathological nervous system. Here we show the role of BAFF-R signaling pathway in the control of neural cell survival. Both B cell–activating factor (BAFF) and its receptor (BAFF-R) are expressed in mouse neurons and BAFF-R deficiency reduces the survival of primary cultured neurons. Although many studies have so far addressed the functional role of BAFF-R on the differentiation of B cells, impaired BAFF-R signaling resulted in accelerated disease progression in an animal model of inherited ALS. We further demonstrate that BAFF-R deficient bone marrow cells or genetic depletion of B cells does not affect the disease progression, indicating that BAFF-mediated signals on neurons, not on B cells, support neural cell survival. These findings suggest opportunities to improve therapeutic outcome for patients with neurodegenerative diseases by synthesized BAFF treatment.

## Introduction

Neurodegenerative diseases, including amyotrophic lateral sclerosis (ALS), Alzheimer's disease (AD) and Parkinson's disease (PD), are incurable and debilitating conditions that result in the progressive degeneration and death of neurons. Despite numerous attempts to identify a treatment strategy for these diseases, there have been no effective therapies to date. Neurotrophic factors (NTFs), such as nerve growth factor (NGF), brain-derived neurotrophic factor (BDNF) and glial cell-line derived neurotrophic factor (GDNF), play pivotal roles in neuronal development and survival and exhibit therapeutic potential in animal models of neurodegenerative diseases [1]. NGF and BDNF also show neurotrophic actions on the cholinergic neurons of the basal forebrain, protecting them against axotomy-induced neurodegeneration and age-related atrophy [2], [3]. Local delivery of NGF to the cholinergic basal forebrain of non-human primates can arrest and even reverse the degeneration of cholinergic neurons that contribute to cognitive decline in AD [4]. GDNF also has robust effects on the survival of dopaminergic neurons in PD [5], [6]. In addition to NTFs, some growth factors, such as vascular endothelial growth factor (VEGF), insulin-like growth factor 1 (IGF1) and hepatocyte growth factor (HGF), have also been shown to exert neuroprotective effects in animal models of ALS [7], [8], [9]. Although these neurotrophic factors and growth factors may have therapeutic potential as neuroprotective factors, most studies have examined these effects using recombinant protein administration and transgenic expression or virus vector-mediated gene transfer. Therefore, it is important to determine if any endogenous factors exert neuroprotective activities in an injured or diseased nervous system.

B cell activating factor (BAFF) is a member of the tumor necrosis factor (TNF) family and is expressed on the surface of monocytes, dendritic cells, neutrophils, stromal cells, activated T cells, malignant B cells and epithelial cells [10]. Cleaved BAFF binds to three different receptors, notably BAFF receptor (BAFF-R), transmembrane activator and calcium modulator and cyclophilin ligand interactor (TACI) and B cell maturation protein (BCMA), that are expressed differentially at various stages of B cell ontogeny [11]. The ligation of BAFF-R by BAFF delivers the potent signals for the survival of B lymphocytes leading to effective humoral immune responses. BAFF transgenic mice develop B cell hyperplasia from the T2 B cell stage, whereas BAFF- and BAFF-R–deficient mice show impaired B cell maturation beyond the T1 stage, decreased immunoglobulin levels, and decreased T cell-dependent and -independent immune responses [12]. These previous findings suggest that, unlike other members of the TNF family, BAFF has its biological activity to a limited repertoire of cell lineages such as B cells. Despite the indispensible function of BAFF in B cell development, a recent study demonstrated BAFF expression in the normal central nervous system (CNS) and some pathogenic lesions of CNS diseases including multiple sclerosis (MS) and primary CNS lymphoma, however it is uncertain whether BAFF contributes to neuronal activity or the disease progression.

In the present work we explore the novel mechanism of neural cell survival by BAFF-R signals using a murine model, in which ablation of the BAFF-R in vivo is combined with the acceleration of neurodegeneration by overexpressing mSOD1. Our findings establish BAFF as a critical member of neurotrophic factors that directs neural cell survival independent of the action for lymphoid cells.

## Materials and Methods

### Ethics Statement

This study was carried out in strict accordance with both the *Guidelines for Animal Experimentation* of the Japanese Association for Laboratory Animal Science and the recommendations in the *Guide for the Care and Use of Laboratory Animals* of the National Institutes of Health. All animal experiments were conducted in accordance with the guidelines of the Animal Care and Use Committee of Research Institute for Microbial Diseases and Immunology Frontier Research Center of Osaka University, who specifically approved this study (Permit number: Biken-AP-H21-28-0). All surgery was performed under sodium pentobarbital anesthesia, and all necessary steps were taken to ameliorate suffering to animals involved in the study.

### Cell culture

Neurons were prepared from cerebral cortices of mouse embryos (E13.5) as previously described [13]. Neurons were maintained in Neurobasal medium (Gibco, MD, USA) containing 2% B27 supplements (Gibco) for 4–7****days before experimentation. Astrocytes were prepared as previously described [13]. Briefly, cells collected from cerebral cortices of newborn mice were plated onto a flask coated with poly-L-lysine (PLL; Sigma) and incubated in media consisting of Dulbecco's modified Eagle's medium (DMEM; WAKO) containing 10% fetal bovine serum (FBS). Nonastroglial cells were removed by shaking on the following day, and the remaining cells were grown further for 3****days, and then astrocytes were removed by trypsinization and replated onto Lab-Tek 8-chamber glass slides coated with PLL.

Mouse Neuro2a cells were originally obtained from the American Type Culture Collection (ATCC cat. no. CCL131). The cells were grown in DMEM containing 10% FBS and penicillin/streptomycin. Murine microglial cell line (6–3 microglial cells) [14] was maintained in Eagle's minimal essential medium, 0.3% NaHCO_3_, 2 mM glutamine, 0.2% glucose, 10 μg/ml insulin and 10% FBS. One ng/ml mouse recombinant GM-CSF was added as a supplement in the culture.

### Immunocytochemistry

Primary cultured neurons (4****days *in vitro*) and Neuro2a cells were fixed in 4% paraformaldehyde in phosphate-buffered saline (PBS; 0.1 M, pH 7.4) for 15 min at room temperature. After washing three times with 0.2% Tween-20 in 0.05 M Tris-buffered saline (TBST, pH 7.2) for 5 min, these cells were maintained overnight in blocking buffer (2% normal rabbit serum for the anti-BAFF antibody and 2% normal donkey serum for the anti–BAFF-R antibody). Fixed cells were incubated overnight at 4°C with the following primary antibodies: a rabbit anti–BAFF-R polyclonal antibody (50 μg/ml; Abcam), goat anti-BAFF polyclonal antibody (15 μg/ml; R&D Systems), and a mouse anti-Map2 monoclonal antibody (1∶200 dilution; Sigma). A rabbit anti-GFAP polyclonal antibody (50 μg/ml; Dako) and goat anti-C4BP polyclonal antibody (15 μg/ml; Santa Cruz Biotechnology) were used as controls to examine BAFF-R and BAFF expression, respectively. The following secondary antibodies were then applied overnight at 4°C: Cy5-conjugated F(ab') 2 fragment rabbit anti-goat IgG (1∶500; Jackson ImmunoResearch Laboratories), Cy5-conjugated F(ab') 2 donkey anti-rabbit IgG (1∶500; Jackson ImmunoResearch Laboratories), and Alexa Flour®488-conjugated anti-mouse IgG (1∶500; Invitrogen). Then, the cells were washed three times in 0.2% TBST for 5 min and mounted with VECTA-SHIELD Mounting Medium containing DAPI (Vector Laboratories). Fluorescence signals were captured with an LSM 510 confocal microscope (Zeiss).

Primary cultured astrocytes were fixed in 4% paraformaldehyde in PBS for 15 min at room temperature. After washing three times with 0.2% TBST for 5 min, these cells were maintained overnight at 4°C in 1% bovine serum albumin. Fixed cells were incubated overnight at 4°C with Alexa Flour®488–conjugated anti-GFAP monoclonal antibody (1∶100 dilution; Cell Signaling Technology). Then, the cells were washed three times in 0.2% TBST for 5 min and mounted with VECTA-SHIELD Mounting Medium containing DAPI (Vector Laboratories). Fluorescence signals were captured with an LSM 510 confocal microscope (Zeiss).

### Immunohistochemistry and lectin staining

Mice were anesthetized with an overdose of pentobarbital (60 mg/kg i.p.) and transcardially perfused with ice-cold 4% paraformaldehyde. The spinal cords were removed, postfixed in the same fixative for 4****h, incubated overnight in 30% sucrose, and embedded in O.C.T. Compound (Sakura Finetek, Tokyo, Japan). Sections were frozen in liquid nitrogen and the blocks were stored at −80°C. Ten-micrometer thick transverse sections of the spinal cords were prepared using a Leica 3050****S cryostat (Thermo Shandon, Inc., Pittsburgh, PA, USA) and then mounted onto Superfrost slides (Matsunami Glass Industries, Ltd., Osaka, Japan).

For immunohistochemistry, the sections were permeabilized with 0.2% TBST for 10 min and pretreated with blocking buffer (2% normal rabbit serum for the anti-BAFF antibody and 2% normal donkey serum for the anti–BAFF-R antibody) for 1****h at RT to block non-specific IgG binding. The following antibodies were used: rabbit anti–BAFF-R polyclonal antibodies (50 µg/ml; Abcam), goat anti-BAFF polyclonal antibodies (50 µg/ml; R&D Systems), mouse anti-SMI32 monoclonal antibody (1∶200; Abcam), and Alexa Flour® 488-conjugated mouse anti-GFAP monoclonal antibody (1∶200; Cell Signaling Technology, Beverly, MA, USA). Rabbit polyclonal IgG (50 µg/ml; Southern Biotechnology Associates, Birmingham, AL, USA) and goat polyclonal IgG (50 µg/ml; Southern Biotechnology Associates) were used as control antibodies for BAFF-R and BAFF expression, respectively. The following secondary antibodies were applied overnight at 4°C: Cy5-conjugated F(ab') 2 fragment rabbit anti-goat IgG (1∶500; Jackson ImmunoResearch Laboratories), Cy5-conjugated F(ab') 2 donkey anti-rabbit IgG (1∶500; Jackson ImmunoResearch Laboratories), and Alexa Flour®488–conjugated anti-mouse IgG (1∶500; Invitrogen). The sections were then washed three times in 0.2% TBST for 5 min and mounted with VECTA-SHIELD Mounting Medium containing DAPI (Vector Laboratories). Fluorescence signals were captured with an LSM 510 confocal microscope (Zeiss).

For lectin staining of the spinal cords, sections were permeabilized with 0.2% TBST for 10 min and then incubated with FITC-conjugated tomato (*Lycopersicon esculentum*) lectin (Sigma) diluted 1∶750 in PBS overnight at 4°C. The sections were washed three times in 0.2% TBST for 5 min and mounted with VECTASHIELD Mounting Medium containing DAPI (Vector Laboratories). The fluorescently labeled sections were examined using a LSM 510 confocal microscope (Zeiss).

For lectin staining of 6–3 microglial cells, cells were fixed in 4% paraformaldehyde in PBS for 15 min at room temperature. After washing three times with 0.2% TBST for 5 min, these cells were incubated with FITC-conjugated tomato (*Lycopersicon esculentum*) lectin (Sigma) diluted 1∶750 in PBS overnight at 4°C. Then cells were washed three times in 0.2% TBST for 5 min and mounted with VECTASHIELD Mounting Medium containing DAPI (Vector Laboratories). Fluorescence signals were captured with an LSM 510 confocal microscope (Zeiss).

### RNA extraction and RT-qPCR analysis

Total mRNA was extracted using the RNeasy Kit (Qiagen, Valencia, CA, USA) according to the manufacturer's recommendations, and the mRNA concentrations were determined spectrophotometrically. cDNA was generated using the SuperScript VILO cDNA Synthesis Kit (Invitrogen) from 100 ng of each RNA sample that had been digested with RNase-free DNase I (Qiagen) according to the manufacturer's recommendations. The synthesized cDNA was amplified using SYBR Premix Ex Taq II (Takara Bio Inc., Shiga, Japan) and the following primer sets: BAFF-Receptor (BAFF-R): 5′-ATGGCTCAGCAGTTCGGTTTG-3′ and 5′- GGCTGGAGTGACAGGTGGTCTTA-3′; BAFF: 5′- TGCCTTGGAGGAGAAAGAGA-3′ and 5′- GGAATTGTTGGGCAGTGTTT-3′; β-actin: 5′- AGCCATGTACGTAGCCATCC-3′ and 5′- TCCCTCTCAGCTGTGGTGGTGAA-3′; and glyceraldehyde-3-phosphate dehydrogenase (GAPDH): 5′-TGTGTCCGTCGTGGATCTGA-3′ and 5′-TTGC TGTTGAAGTCGCAGGAG-3′. A standard thermal cycling program was used for all PCRs: 95°C for 60****s, 40 cycles of 95°C for 5****s, and 60°C for 30****s. The relative expression levels of each mRNA were calculated using the ΔΔCt method, after normalizing to GAPDH or β-actin and relative to bone marrow cells or to the spinal cord of C57BL/6 mouse at 70****days of age.

### Analysis of neuronal survival

The number of viable neurons in primary cultures was evaluated by Map2 staining. Map2-positive neurons were considered viable if they had large (>20 μm) cell bodies, prominent neuritic arborization, and a single long axon-like neurite. The number of neurons was counted microscopically in at least 20 randomly selected fields. Determinations were made for at least three separate cultures.

### Western blot analysis

Western blot analysis was performed as previously described [13]. Samples were lysed with NP40 buffer [PBS, 1% NP-40, 0.5% sodium deoxycholate, and 0.1% sodium dodecyl sulfate (SDS), pH 7.4] containing protease inhibitors (20 μg/ml aprotinin and 1 mM phenylmethylsulfonyl fluoride) and 1 mM sodium orthovanadate. Equal protein levels were resolved on 10% SDS-polyacrylamide gels, which were then transferred onto nitrocellulose membranes (Bio-Rad Laboratories, Hercules, CA, USA). The blots were incubated at 4°C overnight with one of the following primary antibodies: rabbit anti-Akt polyclonal antibody (1∶1000; Cell Signaling Technology), rabbit anti–phospho-Akt (Ser473) polyclonal antibody (1∶1000; Cell Signaling Technology) or mouse anti–β-actin monoclonal antibody (1∶1000; Sigma). The blots were subsequently incubated with the appropriate horseradish peroxidase–conjugated secondary antibodies for 90 min and visualized using SuperSignal West Femto Maxmum Sensitivity Substrate (Thermo Fisher Scientific, Waltham, MA, USA). The image of each band was captured and analyzed using Image Gauge (Fuji Film, Japan).

### Animals

All mice were housed in microisolator cages within a modified pathogen-free barrier facility at the Animal Resource Center for Infectious Diseases, Research Institute for Microbial Diseases, Osaka University. All animals were provided food and water *ad libitum.*

Transgenic mice overexpressing the familial ALS-associated G93A SOD1 mutation (harboring a single glycine to alanine substitution at codon 93) (mSOD1 mice) were obtained from The Jackson Laboratory (strain designated: B6SJL-TgN(SOD1-G93A)1Gurd/J) and backcrossed with C57BL/6 mice for at least 10 generations.

Bcmd1^A/WySnJ^ mice were obtained from The Jackson Laboratory (strain designated: A/WySnJ) and backcrossed with C57BL/6 mice for at least 10 generations.

μMT mice on a C57BL/6 background were obtained from The Jackson Laboratory (strain designated: B6.129S2-Igh-6tm1Cgn/J). In some experiments, mSOD1 mice and μMT mice were crossed to generate mSOD1/μMT mice that are B-cell deficient.

### Weight measurements and hanging-wire test

Mice were weighed and subjected to the hanging-wire test every 3–4****days starting on day 64. For the hanging-wire test, each mouse was placed on the wire lid of a conventional housing cage. The lid was shaken gently to prompt the mouse to hold onto the grid before the lid was swiftly turned upside down. The time period until the mouse let go with both hind limbs was determined. Each mouse was allowed up to three attempts to hold on to the inverted lid for an arbitrary maximum of 90****s and the longest time period was recorded.

### Morphological analysis of the sciatic nerve

Mice were deeply anesthetized, perfused with ice-cold 4% paraformaldehyde, and fixed with 3% glutaraldehyde in PBS buffer, pH 7.4. Sciatic nerve samples were immersed in fixative overnight, rinsed in PBS buffer, and postfixed in 1% osmium tetroxide. After three washes with PBS buffer, the samples were dehydrated in a graded series of ethanol and embedded in Epon (Marivac Canada Inc., Quebec, Canada). Thin sections of the sciatic nerve were stained with toluidine blue and examined under a light microscope. Myelinated axons in the sciatic nerve were counted (n = 3 per group).

### Bone marrow transplantation

Donor bone marrow was obtained from 9–12-week-old *Baffr* ^+/+^ (Ly5.1) or *Baffr* ^m/m^ mice and transplanted into mSOD1/*Baffr* ^m/m^ (Ly5.2) mice within 40****days of birth. mSOD1/*Baffr* ^m/m^ mice were sublethally irradiated (600 rads) and transplanted with bone marrow derived from *Baffr* ^+/+^ or *Baffr* ^m/m^ mice. Briefly, the donor mice were lethally anesthetized and their femurs were removed under sterile conditions. The bone marrow was flushed out of the femurs with Hanks' Balanced Salt Solution (Nacalai tesque, Kyoto, Japan). The hematopoietic cells were successively passed through 18-, 21-, 23-, and 25-gauge needles. The cells were then pelleted at 250****g for 10 min, washed with 5 ml Hanks' Balanced Salt Solution, and resuspended at 7.5×10^7^ cells/ml PBS. Using a 27-gauge needle, a 200 μl aliquot (1.5×10^7^ cells per mouse) was injected i.v. into mSOD1/*Baffr* ^m/m^ mice.

### Flow cytometry

The following antibodies were used: APC-labeled anti-CD45.1 (clone A20; eBioscience, San Diego, CA, USA) and Pacific Blue-labeled anti-Ly5.2 (clone 104; BioLegend, San Diego, CA, USA). Flow cytometry was performed using a FACS Canto™ II with Diva ™ software (Becton Dickinson, Franklin Lakes, NJ, USA). Acquired data were analyzed using FlowJo software (Tree Star, Inc., Ashland, OR, USA).

### Statistics

Differences in survival were determined using Kaplan-Meier survival statistics (log rank test). All other data were analyzed using the Mann-Whitney test and Excel software (Microsoft). Data are expressed as the mean ± SEM. * p<0.05 was considered statistically significant.

## Results and Discussion

**Figure 1:**
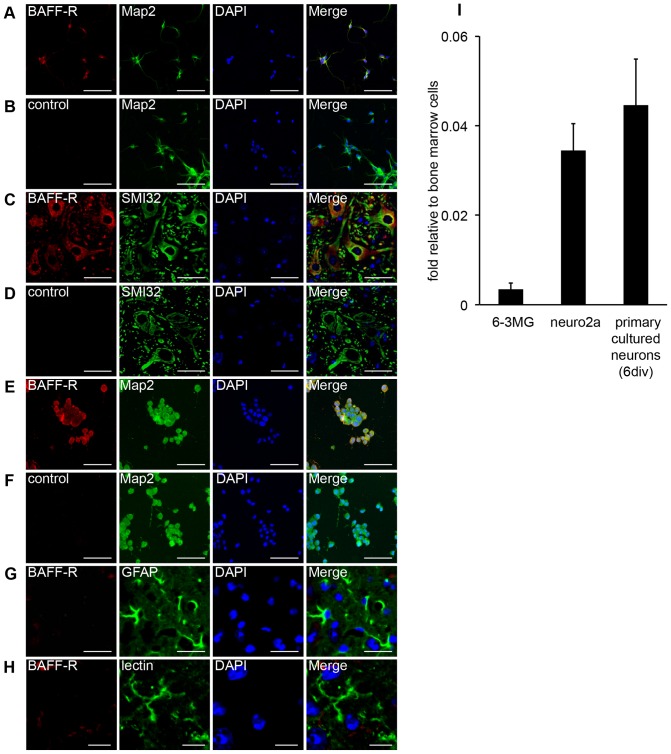
BAFF-R expression in mouse primary cultured neurons, on spinal cord neurons and on Neuro2a cells. (A–F) Primary cultured mouse neurons (A and B), sections of a mouse spinal cord (C and D) and Neuro2a neuroblastoma cells (E and F) were co-stained with Cy5-conjugated anti–BAFF-R antibodies (A, C and E) or control antibodies (B, D and F) and an Alexa488-conjugated anti-microtubule-associated protein 2 (Map2) antibody (A, B, E and F) or anti-Neurofilament H Non-Phosphorylated (SMI-32) antibody (C and D). (G and H) Sections of a mouse spinal cord were co-stained with Cy5-conjugated anti–BAFF-R antibodies and an Alexa488 conjugated anti-GFAP antibody (G) or FITC-conjugated tomato lectin (H). 4′, 6-diamidino-2-phenylindole (DAPI) was also used to stain nuclei. Scale bars represent 100 μm (for panel A, B, E and F), 50 μm (for panel C and D), 25 μm (for panel G), and 10 μm (for panel H) respectively. (I) BAFF-R mRNA expression in 6–3 microglia cells, Neuro2a cells, and primary cultured neurons was examined by quantitative RT-PCR. The data are presented as the mean ± s.d. of samples examined in triplicate.

**Figure 2:**
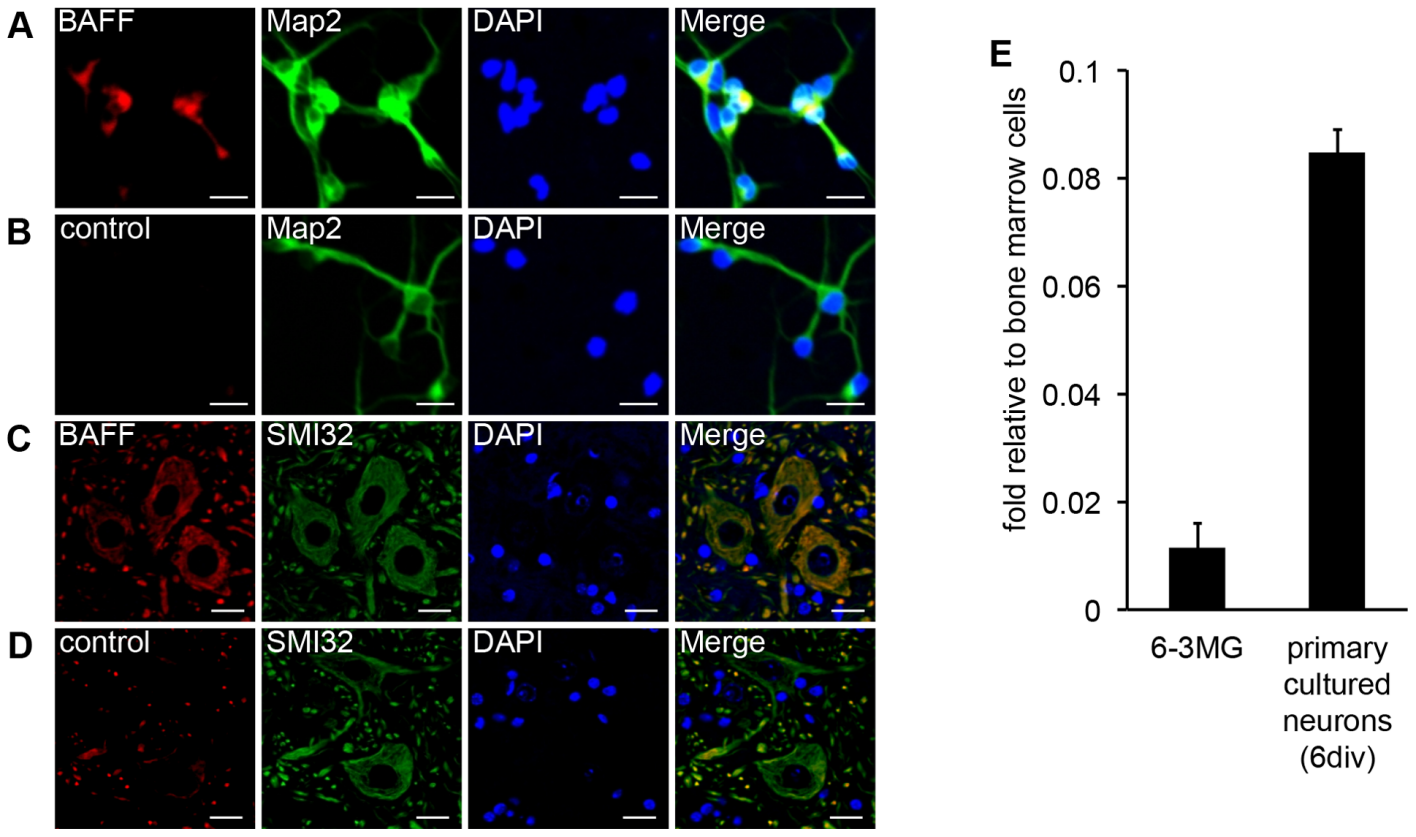
BAFF expression in mouse primary cultured neurons and on mouse spinal cord neurons. (A–D) Primary cultured mouse neurons (A and B) and sections of a mouse spinal cord (C and D) were co-stained with Cy5-conjugated anti-BAFF antibodies (A and C) or control antibodies (B and D) and an Alexa488-conjugated anti-Map2 antibody (A and B) or anti-SMI-32 antibody (C and D). DAPI was also used to stain nuclei. Scale bars represent 20 μm (calculated for each panel). (E) BAFF mRNA expression in 6–3 microglia cells and primary cultured neurons was examined by quantitative RT-PCR. The data are presented as the mean ± s.d. of samples examined in triplicate.

To evaluate BAFF-R and BAFF expression in the CNS, we immunostained cultured murine neurons and murine spinal cords with anti–BAFF-R and anti-BAFF antibodies. BAFF-R was detected in microtubule-associated protein 2 (Map2)–positive primary cultured neurons, Neurofilament H Non-Phosphorylated (SMI-32)–immunoreactive spinal cord motor neurons and Neuro2a cells, a mouse neuroblastoma cell line (**Fig. 1 A–F**). However, an anti–BAFF-R antibody only minimally bound to glial fibrillary acidic protein (GFAP)–positive astrocytes or tomato lectin–positive microglia in the spinal cord. (**Fig. 1 G and H**). Quantitative RT-PCR analysis also showed that BAFF-R mRNA levels were higher in both Neuro2a cells and cultured primary murine neurons than in 6–3MG cells, a microglial cell line (**Fig. 1 I**). Moreover, both immunostaining and quantitative RT-PCR analyses showed that spinal cord and murine primary cultured neurons also express BAFF (**Fig. 2 A–E**). Overall, these data indicate that both BAFF and BAFF-R are expressed in neuronal cells.

**Figure 3:**
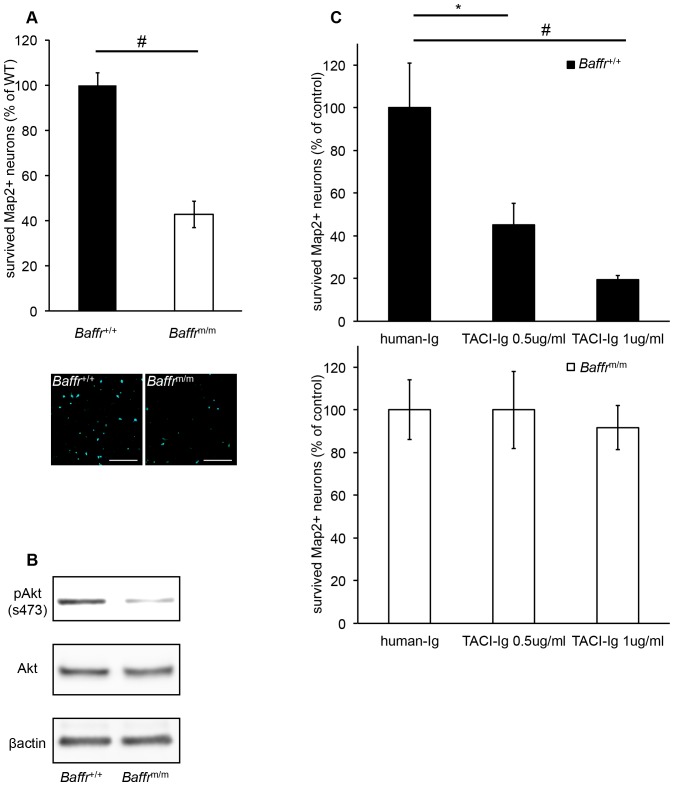
Role of BAFF-R in neuronal survival in vitro. (A) The effect of a BAFF-R deficiency on neuronal survival. Neurons from E13.5 embryos of wild-type and A/WySnJ mice (*Baffr* ^m/m^ mice) were cultured for 7****days under nutrient-limiting conditions, and then cell viability was measured by counting Map2^+^ viable neurons. Representative pictures are shown. Scale bar  = 100 μm. (B) Reduced Akt phosphorylation in *Baffr* ^m/m^ neurons. After 7****days of culture, wild-type and *Baffr* ^m/m^ neurons were assayed for the levels of total and phospho-Akt by immunoblot analysis. β-actin is shown as a loading control. (C) The effects of blocking BAFF-R on neuronal survival. Neurons from E13.5 embryos of wild-type and *Baffr* ^m/m^ mice were cultured with TACI-Ig or control human-Ig for 7****days under nutrient-limiting conditions, and then Map2^+^ viable neurons were counted. Data are representative of three separate experiments. *:p<0.05, #:p<0.01.

Ligation of BAFF-R on B cells has been shown to induce Akt activation and the upregulation of anti-apoptotic genes such as Bcl-xL, leading to B cell survival [12]. Therefore, we investigated the possible contribution of BAFF-R signaling to neuronal survival using A/WySnJ (*Baffr* ^m/m^) mice, which carry a mutation in BAFF-R that results in defective BAFF-R signaling and impaired B cell maturation. Neuronal cells from the brains of either wild-type or *Baffr* ^m/m^ mice were cultured under low-nutrient conditions. The defect in BAFF-R signaling led to a marked reduction in neuronal survival during nutrient withdrawal (**Fig. 3 A**), although there were no differences in cell numbers between the two groups when neurons were cultured under nutrient-rich conditions (data not shown). Concomitantly, phosphorylation of Akt at serine 473 was decreased in neurons in *Baffr* ^m/m^ (**Fig. 3 B**). Next, TACI fused to the Fc portion of human IgG (TACI-Ig), which can prevent murine BAFF from binding to BAFF-R [15], was added to neuronal cultures. Blocking BAFF-R ligation with TACI-Ig inhibited wild-type, but not *Baffr* ^m/m^, neuronal survival in a dose-dependent manner (**Fig. 3 C**). However, TACI-Ig had no effect on the survival of 6–3 microglial cells or primary cultured murine astrocytes (**Figure S1**). Collectively, these results indicate that the functional interaction between BAFF and BAFF-R on neuronal cells contributes to their survival.

**Figure 4:**
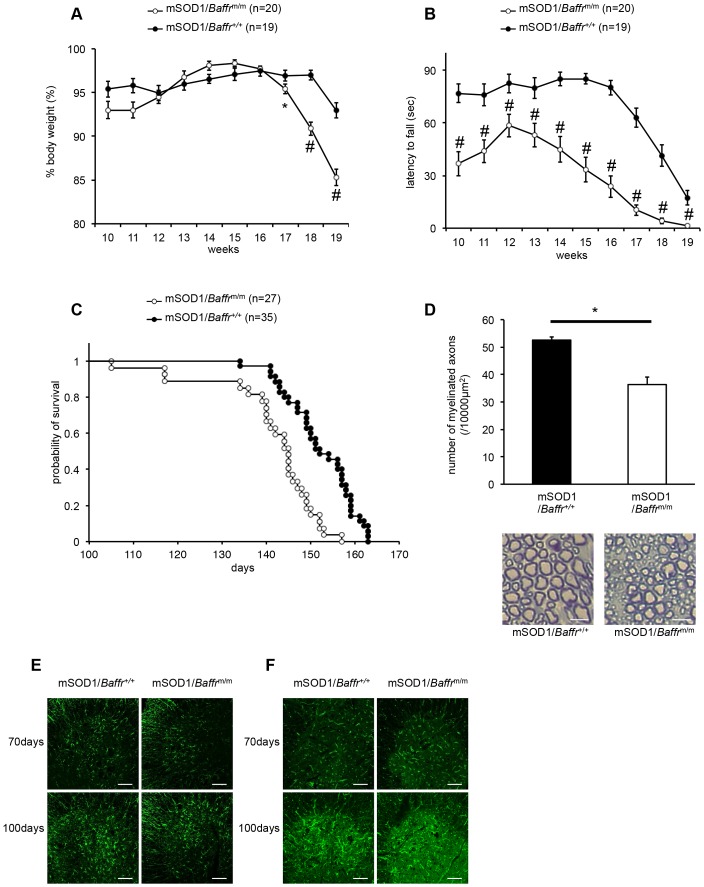
mSOD1/*Baffr* ^m/m^ mice exhibit accelerated disease progression and reduced survival. (A) Changes in the mean body weight of mSOD1/*Baffr* ^m/m^ mice (n = 20) and mSOD1/*Baffr* ^+/+^ mice (n = 19). (B) Time course of motor performance using the hanging-wire test in which mSOD1/*Baffr* ^m/m^ mice performed significantly worse than mSOD1/*Baffr* ^+/+^ mice. (n = 20 and n = 19, respectively) (C) Kaplan-Meier survival curve. Defects in BAFF-R signaling shortened the survival of mSOD1 mice (log rank test for survival, p = 0.0000342) (n = 27 for mSOD1/*Baffr* ^m/m^ mice and n = 35 for mSOD1/*Baffr* ^+/+^ mice) (D) mSOD1/*Baffr* ^m/m^ mice had significantly fewer myelinated axons than control mSOD1/*Baffr* ^+/+^ mice (n = 3 per group). Representative pictures are shown. Scale bar  = 20 μm. (E and F) The number of GFAP-positive or tomato lectin-positive cells in the spinal cord does not differ between mSOD1/*Baffr* ^+/+^ and mSOD1/*Baffr* ^m/m^ mice. Lumbar sections of the spinal cord were stained with an anti-GFAP antibody (E) or with tomato lectin (F). Scale bar  = 100 μm. Data are representative of three animals.

Finally, to determine whether BAFF-R has a neuroprotective role in vivo, we first evaluated the BAFF and BAFF-R expression in the spinal cord of transgenic mice overexpressing the mutant human SOD1 gene (mSOD1 mice), which show not only ALS-like symptoms but also the degeneration of both upper and lower motor neurons [16]. At the age of 70****days, relative expression level of BAFF and BAFF-R in the spinal cords of mSOD1 mice was 0.98±0.19 and 1.72±0.14 respectively. At the age of 130****days, BAFF and BAFF-R expression increased up to 1.83±0.32 and 2.20±0.30 respectively (**Figure S2**), suggesting that BAFF-BAFF-R axis may be involved in the regulation of neuronal survival in mSOD1 mice. Next, we introduced a transgene encoding a mutated human SOD1 into *Baffr* ^m/m^ mice. The resulting mSOD1/*Baffr* ^m/m^ mice displayed significantly accelerated weight loss around 17****weeks of age compared to mSOD1/*Baffr* ^+/+^ mice (**Fig. 4** **A**). In addition, mSOD1/*Baffr* ^m/m^ mice exhibited significant muscle weakness compared to control mice in a hanging-wire test (**Fig. 4** **B**). Furthermore, mSOD1/*Baffr* ^m/m^ mice had shorter life spans compared to control mSOD1 mice (mSOD1/*Baffr* ^+/+^ mice: 152.3±1.3****days (n = 35); mSOD1/*Baffr* ^m/m^ mice: 141.6±2.3****days (n = 27)) (**Fig. 4** **C**). Consistent with these clinical manifestations, mSOD1/*Baffr* ^m/m^ mice had significantly fewer myelinated axons in sciatic nerves than mSOD1/*Baffr* ^+/+^ mice at 75****days of age (**Fig. 4** **D**), although Nissl staining showed no significant difference in the number of motor neurons in the anterior horns (data not shown). On the other hand, there were no differences in the numbers of microglia and astrocytes between mSOD1/*Baffr* ^m/m^ and control mice, although neurodegeneration in ALS often involves neuroinflammation mediated by these cells (**Fig. 4** **E and F**) [17], [18], [19], [20].

**Figure 5:**
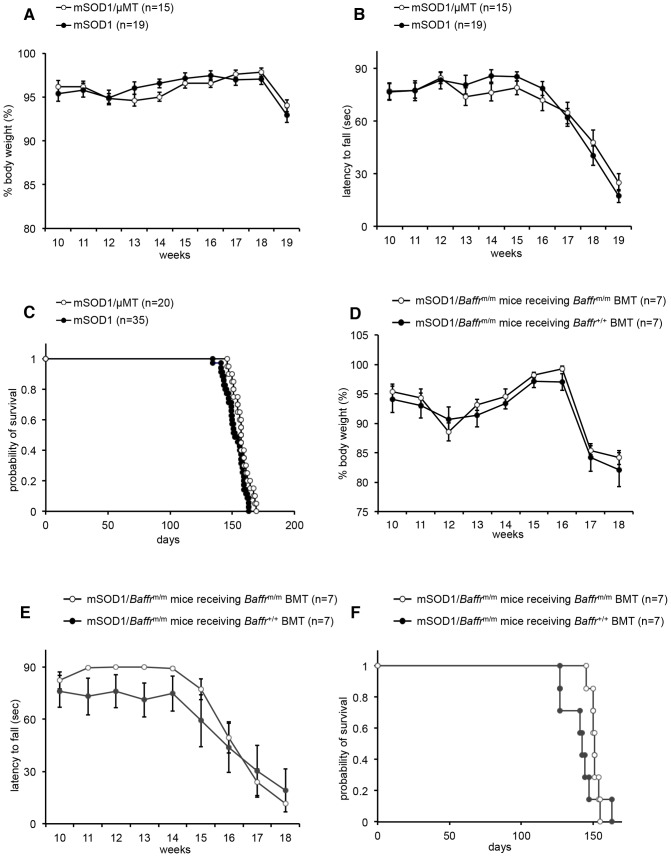
Neither B lymphocytes nor bone marrow-derived cells affect disease progression or the survival of mSOD1 mice. (A–C) The absence of B lymphocytes does not affect body weight (A), motor performance (B), or the survival (C) of mSOD1 mice (log rank test for survival, p = 0.051). In panels A and B, n = 15 for mSOD1/μMT mice and n = 19 for control mSOD1 mice. In panel C, n = 20 for mSOD1/μMT mice and n = 35 for control mSOD1 mice. (D–F) BAFF-R–deficient bone marrow cells do not affect body weight (D), motor performance (E), or the survival (F) of mSOD1 mice. (log rank test for survival, p = 0.284) (n = 7 per group) Data are expressed as the mean ± SEM. *:p<0.05, #:p<0.01.

Other groups previously showed that disease is exacerbated in mSOD1 mice that are deficient for *RAG2* or CD4^+^ T cells [21], [22], suggesting that immune cells contribute to the pathogenesis of ALS. Thus, it is possible that the accelerated disease in mSOD1/*Baffr^m/m^* mice is due to defective mature B cells or a deficiency in BAFF-R on bone marrow-derived cells. However, a recent study showed that B cell-deficient mSOD1 mice (mSOD1/μMT mice) did not show altered mortality or rotarod performance compared with control mice [23]. Indeed, we confirmed that disease progression and severity in mSOD1/μMT mice were indistinguishable from those in control mSOD1 mice (mean survival time of mSOD1/μΜΤ mice: 157.3±1.5****days (n = 20); mean survival time of control mSOD1 mice: 152.3±1.3****days (n = 35)) (**Fig. 5 A–C**), indicating that B cells do not play a protective role in ALS.

To further determine the neuroprotective role of BAFF-R in bone marrow-derived cells, we then transferred bone marrow cells from *Baffr* ^m/m^ or wild-type mice into mSOD1/*Baffr* ^m/m^ mice. The disease progression of mSOD1/*Baffr* ^m/m^ mice reconstituted with bone marrow cells from *Baffr* ^m/m^ mice was comparable to that of mSOD1/*Baffr* ^m/m^ mice reconstituted with wild-type bone marrow cells (mean survival time of mSOD1/*Baffr* ^m/m^ mice receiving *Baffr* ^m/m^ BMT: 150.9±1.2****days (n = 7); mean survival time of mSOD1/*Baffr* ^m/m^ mice receiving *Baffr* ^+/+^ BMT: 141.6±4.7****days (n = 7)) (**Fig. 5 D–F** **and** **Figure S3**). These results collectively suggest that BAFF-R on neuronal cells, but not on bone marrow–derived cells including B cells, contributes to neuroprotection.

Although the role of the BAFF-BAFF-R axis in B cell development and survival has been extensively investigated, their expression and function in other cell lineages have remained unclear. The present study clearly shows that both BAFF and BAFF-R are expressed on neuronal cells and play a role in neuronal survival. Furthermore, experiments with a murine ALS model demonstrated that BAFF-R signals on neurons appear to be necessary for neuroprotection *in vivo*.

The BAFF–BAFF-R axis is necessary not only for the mature B cell survival but also for B cell maturation at later stages of differentiation. BAFF or BAFF-R mutant mice have not previously been shown to have defects in the nervous system. However, it is possible that defects in these mice may be too subtle to detect or that BAFF signals may be compensated by NTFs such as NGF and BDNF, which exert neuroprotective effects and play crucial roles in neuronal development.

Neuronal apoptosis induced by nutrient withdrawal can be prevented by activation of Akt [24]. We also observed reduced phosphorylation of Akt in *Baffr* ^m/m^ neuron in a nutrient withdrawal condition (**Fig. 3B**), suggesting that impaired Akt activation may contribute to accelerated apoptosis of *Baffr* ^m/m^ neurons. Several previous reports have shown the contribution of apoptosis [25], [26] and a protective role of Akt [7], [27], [28], [29], [30] in the motor neuron death in mSOD1 mice. Therefore, it is likely that BAFF-R signal plays a neuroprotective role in neurons of mSOD1 mice by activating Akt and suppressing apoptosis.

Our findings imply that the target spectrum of the BAFF–BAFF-R axis might be much broader than previously thought. Further careful studies may be required to determine their potential role in organs other than the immune and neuronal systems. The present study revealed an unexpected function of BAFF and BAFF-R in the nervous system. Our identification of BAFF as a neuroprotective factor suggests that promoting neuroprotective signaling through the BAFF–BAFF-R axis might be a potential therapeutic target for neurodegenerative diseases such as ALS.

## Supporting Information

Figure S1 **Blocking BAFF binding to BAFF-R did not affect survival of microglia or astrocytes in vitro.** (A) 6–3 microglial cells were treated with TACI-Ig (0.5 μg/ml or 1 μg/ml) or control human IgG (1 μg/ml). After 48****h of incubation, the cells were fixed with 4% paraformaldehyde and stained with FITC-conjugated tomato lectin. DAPI was used to stain nuclei. Scale bars represent 200 μm. (B) Primary cultured astrocytes were treated with TACI-Ig (0.5 μg/ml or 1 μg/ml) or control human IgG (1 μg/ml). After 7****days of incubation, the cells were fixed with 4% paraformaldehyde and stained with Alexa Flour 488-conjugated anti-GFAP antibody. Scale bars represent 200 μm.
(TIF)

Figure S2 **Expression level of BAFF and BAFF-R in the spinal cord of mSOD1 transgenic mice at different age.** mSOD1 transgenic mice were sacrificed at the age of 70 and 130****days, and RNA was isolated from homogenized flash-frozen spinal cords. BAFF and BAFF-R expression level was analyzed by RT-qPCR experiments. n = 4 for mSOD1 mice at 70****days of age and n = 3 for mSOD1 mice at 130****days of age. The data are presented as the mean ± s.e.m.
(TIF)

Figure S3 **A flow cytometric profile of peripheral blood lymphocytes from mSOD1/** ***Baffr*** **^m/m^ mice after bone marrow transplantation.** mSOD1/*Baffr* ^m/m^ mice expressing the Ly5.2 marker were subjected to bone marrow transplantation with bone marrow cells from *Baffr^+/+^* mice expressing the Ly5.1 marker, after mild irradiation (600 rads). Chimerism and peripheral reconstitution were analyzed by flow cytometry eight weeks after bone marrow transplantation. The percentages of gated populations are shown.
(TIF)
