# Supplementary figures and images for: BAFF Controls Neural Cell Survival through BAFF Receptor
Source: PLoS One. 2013 Jul 29;8(7):e70924. doi: 10.1371/journal.pone.0070924 (PMC3726595; doi:10.1371/journal.pone.0070924)

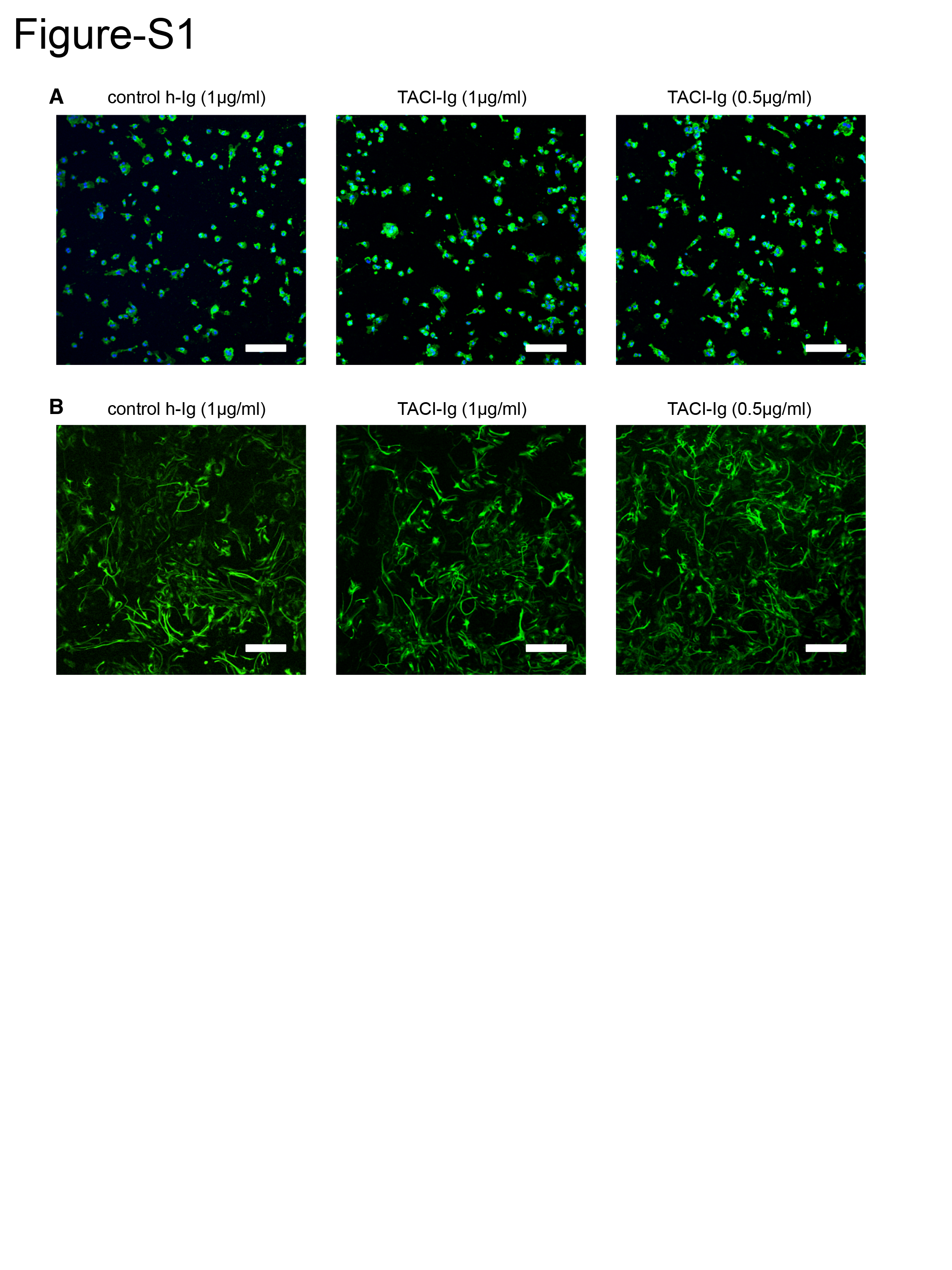

Supplement: Figure S1 — Blocking BAFF binding to BAFF-R did not affect survival of microglia or astrocytes in vitro. (A) 6–3 microglial cells were treated with TACI-Ig (0.5 μg/ml or 1 μg/ml) or control human IgG (1 μg/ml). After 48h of incubation, the cells were fixed with 4% paraformaldehyde and stained with FITC-conjugated tomato lectin. DAPI was used to stain nuclei. Scale bars represent 200 μm. (B) Primary cultured astrocytes were treated with TACI-Ig (0.5 μg/ml or 1 μg/ml) or control human IgG (1 μg/ml). After 7days of incubation, the cells were fixed with 4% paraformaldehyde and stained with Alexa Flour 488-conjugated anti-GFAP antibody. Scale bars represent 200 μm. (TIF) [file pone.0070924.s001.tif]

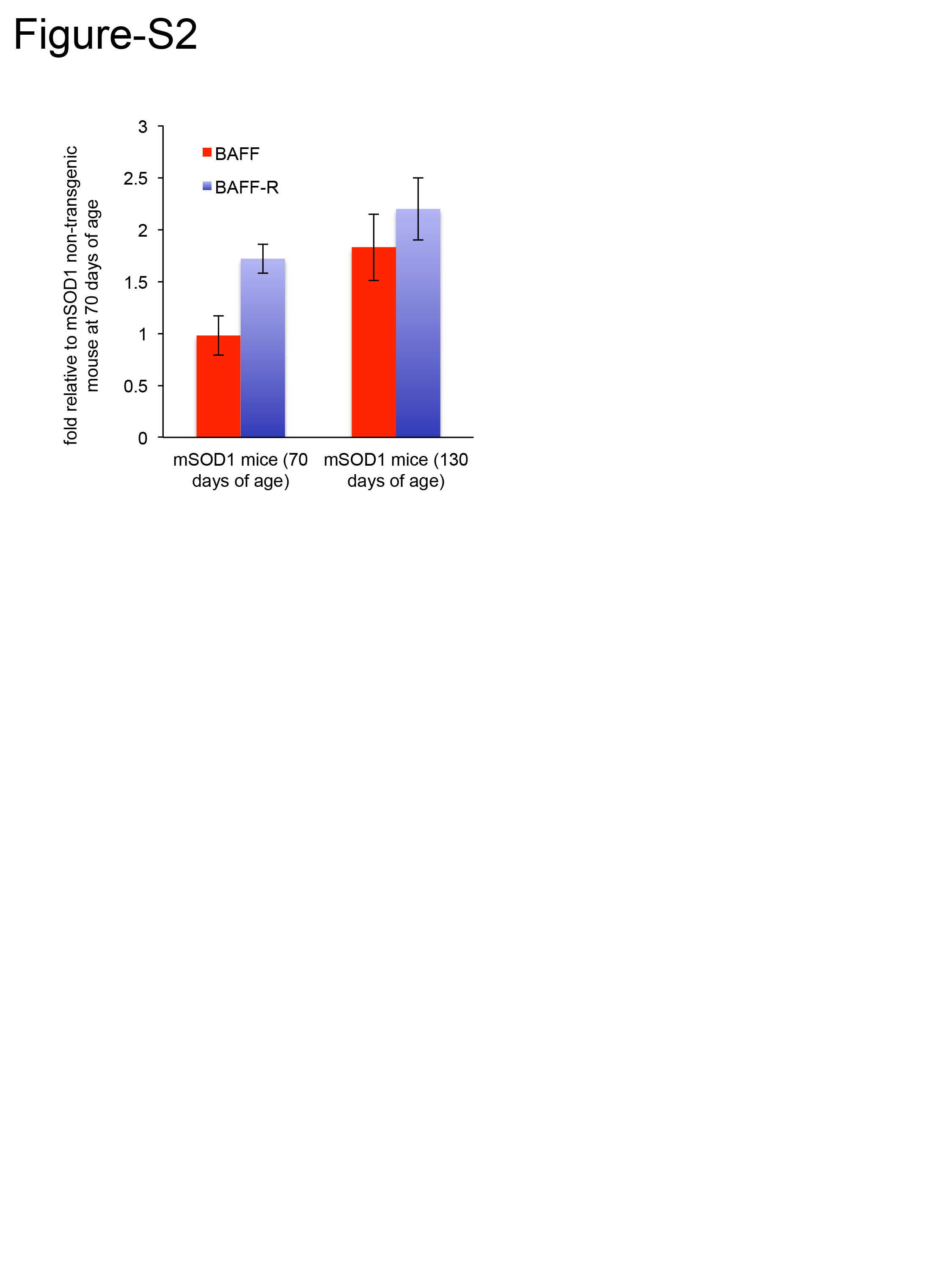

Supplement: Figure S2 — Expression level of BAFF and BAFF-R in the spinal cord of mSOD1 transgenic mice at different age. mSOD1 transgenic mice were sacrificed at the age of 70 and 130days, and RNA was isolated from homogenized flash-frozen spinal cords. BAFF and BAFF-R expression level was analyzed by RT-qPCR experiments. n = 4 for mSOD1 mice at 70days of age and n = 3 for mSOD1 mice at 130days of age. The data are presented as the mean ± s.e.m. (TIF) [file pone.0070924.s002.tif]

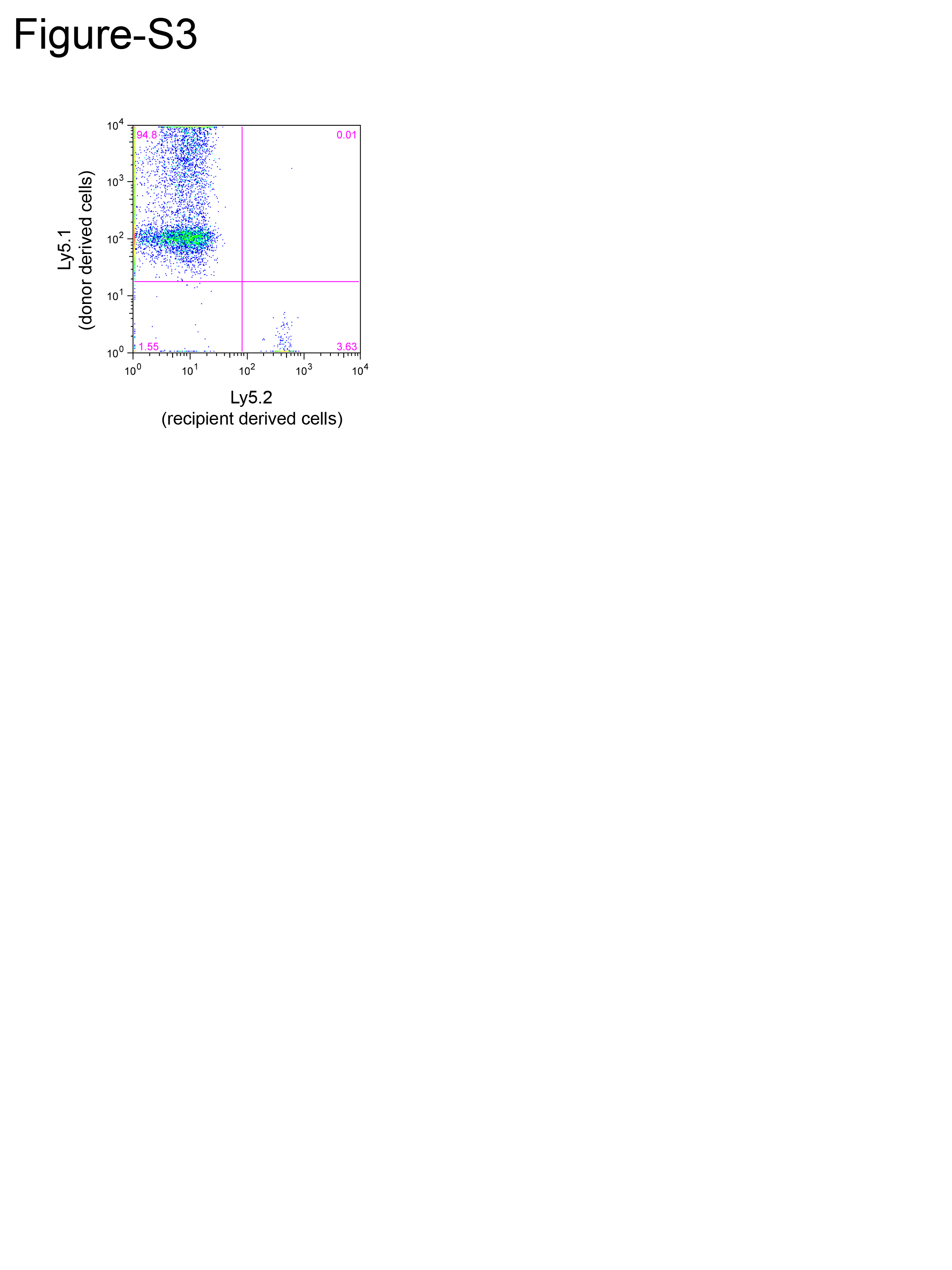

Supplement: Figure S3 — A flow cytometric profile of peripheral blood lymphocytes from mSOD1/ Baffr m/m mice after bone marrow transplantation. mSOD1/Baffr m/m mice expressing the Ly5.2 marker were subjected to bone marrow transplantation with bone marrow cells from Baffr+/+ mice expressing the Ly5.1 marker, after mild irradiation (600 rads). Chimerism and peripheral reconstitution were analyzed by flow cytometry eight weeks after bone marrow transplantation. The percentages of gated populations are shown. (TIF) [file pone.0070924.s003.tif]
